# Cardiovascular involvement during COVID-19 and clinical implications in elderly patients. A review

**DOI:** 10.1016/j.amsu.2020.07.054

**Published:** 2020-08-05

**Authors:** Claudio Napoli, Isabella Tritto, Giuditta Benincasa, Gelsomina Mansueto, Giuseppe Ambrosio

**Affiliations:** a Clinical Department of Internal Medicine and Specialistic Units, Department of Advanced Medical and Surgical Sciences (DAMSS), University of Campania “Luigi Vanvitelli”, Naples, Italy; b IRCCS SDN, Naples, Italy; c Cardiology and Cardiovascular Pathophysiology, Azienda Ospedaliero-Universitaria “S. Maria Della Misericordia”, Perugia, Italy

**Keywords:** COVID-19, SARS-Cov 2, ACE2, Inflammation, Myocarditis, Vasculitis, Heart failure, Troponin, Cytokine release syndrome, Cardiovascular drugs

## Abstract

SARS-CoV-2 betacoronavirus is responsible for the Corona Virus Disease 2019 (COVID-19) which has relevant pathogenic implications for the cardiovascular system. Incidence and severity of COVID-19 are higher in the elderly population (65 years and older). This may be due to higher frequency of comorbidities, but increased frailty and immunosenescence linked with aging may also contribute. Moreover, in elderly individuals, SARS-CoV-2 may adopt different molecular strategies to strongly impact on cardiac aging that culminate in exacerbating a pro-inflammatory state (cytokine storm activation), which, in turn, may lead to pulmonary vascular endothelialitis, microangiopathy, diffuse thrombosis, myocarditis, heart failure, cardiac arrhythmias, and acute coronary syndromes. All these events are particularly relevant in elderly patients, and deserve targeted cardiovascular treatments and specific management of repurposed drugs against COVID-19. We discuss current evidence about the cardiovascular involvement during COVID-19, and elaborate on clinical implications in elderly patients.

## Highlights

• SARS-CoV-2 infection has relevant pathogenic implications for the heart, mainly in elderly patients.
• Common cardiometabolic comorbidities and aging strongly contribute to higher frequency and severity of disease in elderly.
• SARS-CoV-2 may directly and indirectly damage the heart leading to multi-organ failure and death.
• Network-oriented analysis are providing novel insight about SARS-CoV-2 pathogenic mechanisms and putative drug targets.

## 1 Introduction

Aging and senescence are complex processes [1,2]. Aging is associated with increased frailty of the body functions, particularly of the cardiovascular system [3,4], increased oxidative stress [5], and decreased endogenous protective mechanisms [[6], [7], [8]]. Moreover, increasing age is associated with reduced efficiency of thrombolysis [9], lower protection afforded by physical exercise against myocardial ischemia [7,10], as well as increased incidence of heart failure [11].

Many pathogens are more aggressive and prevalent in the elderly population [12]. SARS-CoV-2 betacoronavirus (large RNA virus) is the virus responsible for Corona Virus Disease 2019 (COVID-19), a respiratory infection, with relevant effects on the cardiovascular system [13,14]. Furthermore, patients with pre-existing cardiovascular conditions represent a relevant subset of elderly patients with symptomatic form of the disease and consequent worse outcomes [13,15]. Table 1 shows clinical studies regarding COVID-19 that have stratified cardiac vs non-cardiac patients according to increasing age and gender [[16], [17], [18], [19], [20]].

**Table 1:** Cardiac patients stratified by aging in COVID-19.

Age as univariable parameter (with and without cardiovascular disease)								
Hospital	Type of study	Sample size	Median age	Gender	Age and cardiac/non cardiac association	Age, cardiac comorbidities	Age and mortality	Ref.
Renmin Hospital of Wuhan University, Wuhan, China (from Jan 20 to Feb 10, 2020)	Cohort study, single center	416 hospitalized patients	64ys	50.7% of patients were female with a less proportion of prior cardiac injury vs men (46.3% vs 51.8%)	19.7% of patients had cardiac injury and were older with respect to patients without cardiac injury (median age, 74 vs 60 ys)	19.7% of patients had more comorbidities, e.g., hypertension (59.8% vs 23.4%;), and higher mortality (51.2% vs 4.5%) vs patients without cardiac injury	Elevation of high-sensitivity troponin I (TnI) levels	[16]
Seventh Hospital of Wuhan City, China (from Jan 23 to Feb 2020)	Retrospective, singlecenter case series	187 hospitalized patients	58ys	Patients with elevated TnT levels had a higher proportion of men vs normal TnT levels (65.4% vs 42.2%)	Patients with elevated TnT levels were older vs patients with normal TnT levels (mean age 71 vs 53 ys)	Patients with elevated TnT levels had significantly higher rates of comorbidities including hypertension (63.5% vs 20.7%), CHD (32.7% vs 3.0%), cardiomyopathy (15.4% vs 0) and COPD (7.7% vs 0)	The mortality during hospitalization was higher in older patients with cardiac injury and elevated TnTs (69.44%)	[17]
Civil Hospitals of Brescia, Lombardy, Italy (from 4 to March 25, 2020)	Cohort study, single center	99 consecutive hospitalized patients	67ys	Patients with cardiac disease had a higher proportion of men (85%); Among non survivors, 89.5% were male	n = 53 patients with cardiac disease and n = 46 without cardiac disease; Patients with cardiac disease were older (mean age 68 ys)	Compared with survivors, non survivors had a history of HF (63.2% vs 26.5%), diabetes (47.4% vs 20.6%), and CHD (47.4% vs 20.6%)	Compared with survivors, non survivors were older (mean age 65 ys)	[18]
**Age as independent factor**								
Hospital	Type of study	Sample size	Median age	Gender	Age and mortality	Mortality rate and laboratory findings		Ref
Wuhan Union Hospital, China (from Jan 10 to Feb 22, 2020)	Cohort study	93 consecutive hospitalized patients	51ys	44% patients were male, and 60% of them died with respect to female (40%)	Non survivors were significantly older (69.0ys ± 10.5 ) than survivors (43.7ys ± 13.1)	The mortality rate increased with chest CT scores, neutrophil-to-lymphocyte ratio, neutrophil percentage, D-dimer level, lactate dehydrogenase level and erythrocyte sedimentation rate, while negatively correlated with the lymphocyte percentage and lymphocyte count		[19]
135 from Jinyintan Hospital and 56 from Wuhan Pulmonary Hospital	Retrospective, cohort study	191 patients	56ys	Most patients were male (62%)	Non survivors were significantly older (69.0ys ± 10.5) than survivors (43.7ys ± 13.1) and the most were male (70%)	Older age, D-dimer levels greater than 1 μg/mL, and higher SOFA score on admission were associated with higher in-hospital death		[20]

Abbreviations: CHD: Coronary Heart Disease; COPD: Chronic Obstructive Pulmonary Disease; HF: Heart Failure; SOFA: Sequential Organ Failure Assessment.

Here, we describe the pathogenic events occurring in the cardiovascular system of elderly patients affected by COVID-19 and their possible clinical management.

## 2 Cardiovascular damage induced by COVID-19

### 2.1 Heart involvement

**Fig. 1:**
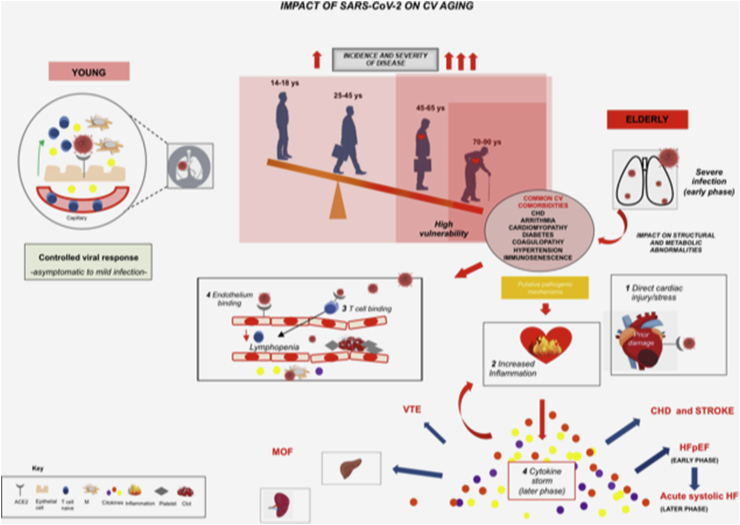
Impact of SARS-CoV-2 infection on cardiovascular aging. Pathogenic mechanisms by which viral infection may have detrimental effects on the cardiovascular system, such as direct cardiac injury/stress, direct T cell and endothelium binding, and increased inflammation. By these ways, SARS-Cov-2 might early impact on prior structural heart and metabolic abnormalities characterizing the elderly patients. In the later stages of infection, the detrimental cytokine storm can in turn exacerbate the pro-inflammatory state leading to cardiovascular events and multiorgan failure.

Patients suffering of COVID-19 can present cardiac arrhythmias, heart failure, myocarditis, pericarditis, and vasculitis [21,22] with increased troponin release [13,23,24]. The structure of the SARS-CoV-2 binding site shows enhanced affinity for the angiotensin I converting enzyme 2 (ACE2) receptor which is expressed mainly in the lung and other tissues including vascular endothelial cells [25,26]. Patients with cardiovascular diseases are more prone to respiratory virus illness, thus constituting a high-risk group [[27], [28], [29]]. Viral entry into the myocardium and artery vessels through ACE2 receptor can enhance the risk for myocardial injury and inflammatory response (Fig. 1) [30]. However, it remains to be established whether a higher rate of cardiac injury with increasing age is due to a direct viral injury or an overwhelming immunological response within myocardium and microvasculature, or both [[31], [32], [33], [34]].

ACE2 has been confirmed as the SARS-CoV-2 internalization receptor [26,35,36] together with the TMPRSS2 membrane protease that primes the spike S protein of the virus [37] and significantly enhances viral shedding [38]. In heart failure, expression of ACE2 is downregulated, though its function may be upregulated [39], reflecting increased ACE2 activity. Relevantly, ACE2 is an immune modulator interacting with macrophages in the setting of inflammation [40], and it also reduces the levels of angiotensin II which is pro-inflammatory and pro-oxidant, while increasing angiotensin 1-7 concentrations that exert opposite effects [41]. In terms of cardiac manifestations of COVID-19, the spectrum is similar to a general viral myocarditis [42].

### 2.2 Inflammatory events

Infected subjects with robust immune response can manifest acute myocarditis with heart failure, accompanied by elevations of cytokines and inflammatory cell infiltration of the heart [43]. Lymphopenia occurs in over 80% of patients with marked reduction in circulating levels of CD4^+^ and CD8^+^ T lymphocytes, and relative prevalence of mononuclear cells (monocytes and macrophages) in target injury tissues [43] (Fig. 1). Thus, decreased capacity to phagocytose apoptotic cells by senescent macrophages may induce a vascular pro-inflammatory state. The immune system imbalance is typical of aging and is exacerbated upon SARS-CoV-2 infection leading to further depletion of CD4^+^T cells and macrophage response [34]. Thus, elderly subjects may have a natural tendency to have reduced virus clearance, which in turn generates an inappropriate *cytokine storm*, with inadequate immune response and immunologic memory.

### 2.3 Endothelialitis

Another pathogenetic hallmark of COVID-19, which distinguishes it from most infectious diseases, is the ability of the virus to directly infect endothelial cells and cause their perturbation and injury, leading to a pro-inflammatory status as well as plasminogen activator release (responsible for very high levels of D-dimers) and high molecular size multimeric von Willebrand factor levels which cause thrombotic microangiopathy [30,43]. In a very recent study, Ackermann et al. [44] have analyzed the up-regulation of genes regulating the “*intussusceptive angiogenesis*” process by using direct multiplexed measurements in the autopsy of COVID-19 patients compared to H1N1 and healthy lungs. An “attachment of lymphocytes to the vascular wall”, a feature called “endothelialitis” or “endothelitis”, has been initially documented in liver biopsies [45] and arteriosclerosis in heart transplant recipients [46]. COVID-19 induces reduction of lymphocytes, mainly CD4^+^ and CD8^+^ T cells, and secondary viral sepsis [47]. Viral particles and SARS-CoV2 RNA have been detected in T lymphocytes from peripheral blood, spleen, and lymph nodes, suggesting that SARS-CoV2 infects T cells directly [47]. The master gene hypoxia-inducible factor (HIF) regulates genes of adaptive response including modulators of inflammation but also of angiogenesis suggesting novel potential therapeutic strategies [48]. Further studies will determine whether differences in angiogenesis and endothelialitis represent distinct points in a similar process of pneumonia or a *true endotype* occurring selectively during the *cytokine storm* seen in COVID-19 patients (Fig. 1).

Patients with clinical evidence of vasculitis, or laboratory indicators of thrombotic risk, such as higher D-dimer levels, should be considered with no doubt for early treatment with antithrombotic drugs. Moreover, many of these patients should be considered also for full anticoagulation, such as heparin, depending on individual risk versus benefit, especially in elderly individuals [49].

## 3 Clinical implications in the care of geriatric patients

Current epidemiology suggests that about 80% of COVID-19 patients have mild symptoms, around 45% have severe symptoms requiring hospitalization, while 5% become critically ill requiring mechanical ventilation [[50], [51], [52]]. The differences in response are likely the result of degree of viral load, host immune response, and, more relevantly, age of the patient and presence of comorbidities, mainly hypertension, diabetes, and thrombotic events [53,54] (Table 1) (Fig. 1). Indeed, aging shows a remarkable impact on both cardiac structure and vasculature leading to pathological abnormalities, such as endothelial dysfunction, left ventricular (LV) hypertrophy, impaired LV diastolic function, as well as increased arterial thickening and stiffness, contributing to hypertension, coronary heart disease (CHD), myocardial infarction (MI), and stroke, until the onset of heart failure [55]. Thus, SARS-CoV-2 infection might be cause of worsening of clinical conditions in elderly patients by impacting on prior structural heart diseases through different putative molecular mechanisms, such as direct myocardial and endothelial binding, T cell death (lymphopenia), and exacerbated inflammation leading to complications in micro- and macrovascular domains, multiorgan dysfunction (MOF), and death (Fig. 1). In elderly patients with potential dysfunctional immune responses there are early warning signals, such as lymphopenia, troponin release, elevated BNP, rising inflammatory markers such as cRP, IL-1β and IL-6 [56]. These aging patients should be followed closely and monitored for early evidence of organ failure, with efforts made to restore immunosenescence and cellular-mediated response [34]. If viral proliferation is still continuing, strategies to attenuate the virus may be critical. However, the intervention will need to be instituted early, before the immune amplification process is fully underway.

Furthermore, obesity which is common among elderly subjects represents another independent risk factor linked to severe COVID-19 and its complications [57]. Indeed, obesity triggers a chronic pro-inflammatory state characterized by elevated levels of IL-6, CRP, and adipokine which are further increased upon viral infection leading to the dangerous cytokine storm [57]. Besides, elderly patients with chronic obstructive pulmonary disease (COPD) were at higher risk of developing severe and critical clinical forms of COVID-19 resulting in higher mortality rate [58].

Whereas the release of troponin is relatively modest during COVID-19, this may be an indication of either viral or immune-mediated cardiac injury. The release of “danger signals” from myocytes in patients with heightened immune response can further amplify myocardial injury [[16], [17], [18],^23^,^59^]. Thus, patients with cardiac injury and/or stress marker release warrant more careful monitoring and institution of cardioprotective agents to minimize ongoing damage [[16], [17], [18],^23^,^59^]. In patients with suspected acute myocarditis, inflammatory cardiomyopathy, and pericarditis, appropriate investigations, such as magnetic resonance imaging where feasible and safe to perform, can be helpful [60,61]. Moreover, electrocardiographic abnormalities reflecting cardiovascular dysfunction have been reported in elderly patients affected by severe COVID-19 [62]. Furthermore, chest X-ray plays a relevant role in the early diagnosis and treatment for patients with suspected or confirmed COVID-19 [63] as well as lung computed tomography is recommended during hospitalization to monitor COVID-19 and heart failure [64]. A great attention should also be devoted to pulmonary and cardiac rehabilitation of COVID-19 survivors [65].

## 4 Therapeutic implications

### 4.1 Cardiovascular drugs during COVID-19

Multiple clinical trials are currently in progress using drug interventions to treat COVID-19 [66] and many others are under development. Down-regulation of ACE2 with viral infection may predispose to relatively unopposed angiotensin II effects, such as hypertension, enhanced inflammation, and thrombosis [41]. Most patients with cardiovascular diseases are treated with ACE inhibitors (ACEi) or angiotensin II receptor blockers (ARBs) therapy [67]. Since both ACEi and ARBs could significantly increase the amount of cardiac ACE2 mRNA, it has been hypothesized that these drugs might impact the cardiac transcriptome, contributing to the aggressive course of COVID‐19 [68]. However, major cardiology scientific associations, such as ESC Hypertension Council, HFSA, ACC, and AHA have rejected this correlation hypothesis concluding that there is no significant evidence to stop therapeutic treatment with these drugs [61,69]. Indeed, as there are no definitive data on the risk versus benefit of ACEi or ARBs in COVID-19, most professional organizations have recommended continuation of renin-angiotensin system inhibitors (RASi) in patients who have been prescribed them [61,69]. Actually, emerging data suggests that benefits may outweigh risks in patients with COVID-19 and hypertension when prescribed ACEi or ARBs [[67], [68], [69], [70]]. Ongoing randomized trials will help providing definitive answers. In patients with heart failure, appropriate medications, including RASi , should be considered [61]. The risk/benefit of statin use is still controversial in patients affected by COVID-19, except for patients with diabetes, history of stroke or CHD , and familial hypercholesterolemia [71,72]. However, a molecular docking study has suggested that statins may act as SARS-CoV-2 Mpro inhibitors (a protein of the viral envelope); clinical investigations are needed to clarify whether statins could exert beneficial effects in the treatment of COVID-19 [73].

### 4.2 Repurposed drugs

Low resting heart rate (RHR) is associated with health and longevity, and conversely, high RHR associated with disease and adverse events [[74], [75], [76], [77], [78]]. Advances in technology have provided new insights into genetic factors related to RHR as well as insights into whether elevated RHR is a risk factor or risk marker [79]. Besides, genome-wide association studies in the general population using Mendelian randomization have demonstrated a causal link between RHR and longevity [74]. Since the COVID-19 pandemic was a global disaster, many repurposed drugs are being tested. Chloroquine or hydroxychloroquine may interfere with cellular endocytosis of the virus [80]; however, these drugs may prolong QT and, thus, ECG monitoring needs to be performed [81]. In this regard, data from Wuhan, China, has indicated that arrhythmias and acute cardiac injury are among the most prevalent heart complications in hospitalized patients (16.7% and 7.2%, respectively) [50]. Recent studies have questioned the benefit of hydroxychloroquine or chloroquine in the treatment of patients affected by COVID-19 owing to serious side effects disturbing the heart rhythm when prescribed at high doses or combined with azithromycin [82,83]. Abnormal QT prolongation, defined as a prolongation to a QTc >500 msec, has been documented for 10%–25% of COVID-19 patients treated with hydroxychloroquine/azithromycin. Many of these patients were elderly and had comorbidities increasing the risk of drug-induced long QTS. Physicians should pay particular attention to elderly females, patients with structural heart disease, baseline QT interval on ECG, concomitant use of other QT-prolonging drugs, potassium or magnesium abnormalities, and recorded episodes of bradycardia [84]. However, only results from ongoing randomized trials will provide definitive answers raising the issue of needing more rigorous data during COVID-19 pandemic [85].

The ability to restore immune balance, with approaches such as type I interferon, immunoglobulin, and recovered serum, may be considered. Among the most important cells of primary immunity that causes cytokine storm there are mast cells that reside in all vascularized tissues generating pro-inflammatory cytokines such as IL-1 and IL-6 [[86], [87], [88], [89]]. Remarkably, suppression both of IL-1 and IL-6 have been shown to have a therapeutic effect in patients with immune system suppression and advanced form of COVID-19 [[86], [87], [88], [89]]. Anti-inflammatory strategies, whether *anti*-IL-1 or *anti*-IL-6 approaches (such as tocilizumab), will be determined in randomized trials. Preliminary studies indicated some beneficial effects of tocilizumab, a IL-6 receptor antagonist, in the clinical management of COVID-19 [[90], [91], [92]].

### 4.3 Antivirals and vaccine development

Many antivirals have been administered to COVID-19 patients. A recent trial with lopinavir-ritonavir gave disappointing results [93]. Viral proliferation can be blocked at multiple stages, including inhibition of RNA polymerase with remdesivir. Preliminary data seems to indicate that remdesivir was superior to placebo in shortening the time to recovery in adults hospitalized with COVID-19 [94,95]. Among the regions of the S protein (S1, S2, RBD), RBD is one accessible target for vaccine. Nevertheless, RBD is a genomic variable region [96] with a risk for immunologic “escape” [97]. The immunogenic role of the N protein, a more conserved region of the genome, is still poorly understood in immune response to SARS-CoV-2 both in adult and elderly patients [96,97]. Vaccine development is the best successful approach for reducing viral diseases, including COVID-19. Until now, there are 70 active clinical trials related to a SARS-CoV2 vaccine (see ClinicalTrials.gov). Overall, people who have more than 60 years are believed to have lesser protective effects by vaccines due to age-related decline in immune function [98]. Thus, even when a vaccine for SARS-CoV2 will become available, particular attention will be due to elderly subjects.

## 5 Concluding remarks

Elderly patients, particularly those with underlying structural heart disease, are the highest risk group for severe COVID-19 and its complications. Currently, diagnostics and personalized therapy of severe and critically ill patients remain the major challenges. In the early stage of the disease, we need to avoid “fever phobia” [99]. The greatest benefit would be seen when drugs are administered 30 h before severe symptom onset, especially in elderly subjects [100]. In this regard, network medicine [101,102] and precision medicine [103] would be helpful in the clinical evaluation of these patients. Cardiovascular dysfunction, cytokine storm, and lymphocytopenia are hallmarks of COVID-19 associated with a poor clinical outcome; however, the precise mechanistic basis is unknown. Despite several clinical trials are ongoing to assess the effectiveness of repurposed drugs, no definitive therapies are currently available. Only dedicated prospective trials in patients of different ages will provide definitive answers to the precise role of aging in COVID-19 therapy. Among gender-related differences, females seem to have a higher susceptibility to SARS-CoV-2 but lower severity and mortality [104,105]. However, further investigations are required to better understand the gender-specific difference in the development of COVID-19. Much advance in the knowledge of SARS-CoV-2 pathogenic mechanisms is coming from network-oriented analysis which provides maps of human proteins interacting with SARS-CoV-2 proteins suggesting putative candidate repurposed drugs and potential combination therapies [106]. The elderly population residing in long-term care facilities suffering from multiple comorbidities [107] and thus more prone to COVID-19 related sequelae. The Italian National Institute of Health COVID-19 mortality group showed that elderly adults (≥65 years) had a higher number of comorbidities compared to those aged <65 years [108]. Interestingly, the prevalence of CHD, atrial fibrillation, heart failure, hypertension, and COPD was higher in older patients (≥65 years). Thus, cardiovascular aging needs to be evaluated carefully in the context of COVID-19.

## Ethical approval

Not applicable.

## Source of funding

This research did not receive any specific grant from funding agencies in the public, commercial, or not-for-profit sectors.

## Author contribution

CN, IT, GB, GM: This Authors contributed to the design and implementation of the review and to the writing of the manuscript.

CN and GA: This Authors provided critical feedback and contributed to the writing of the manuscript.

## Registration of research studies

Not applicable.

## Guarantor

GA is the Guarantor of this manuscript.

## Consent

Not applicable.

## Provenance and peer review

Not commissioned, externally peer reviewed.

## Declaration of competing interest

All the Authors declare no conflict of interest.
